# Long-Term Repeatable In Vivo Monitoring of Amyloid-β Plaques and Vessels in Alzheimer’s Disease Mouse Model with Combined TPEF/CARS Microscopy

**DOI:** 10.3390/biomedicines10112949

**Published:** 2022-11-16

**Authors:** Ziyi Luo, Hao Xu, Soham Samanta, Renlong Zhang, Guoquan Luo, Yiming Wang, Liwei Liu, Xiaoyu Weng, Jun He, Changrui Liao, Yiping Wang, Bingang Guo, Junle Qu

**Affiliations:** 1 Shenzhen Key Laboratory of Photonics and Biophotonics, College of Physics and Optoelectronic Engineering, Shenzhen University, Shenzhen 518060, China; 2 Key Laboratory of Optoelectronic Devices and Systems of Ministry of Education and Guangdong Province, College of Physics and Optoelectronic Engineering, Shenzhen University, Shenzhen 518060, China; 3 Holokook Co., Ltd. Shenzhen, Shenzhen 518052, China

**Keywords:** long-term imaging, Alzheimer’s disease (AD), amyloid-β (Aβ), cranial window, two-photon excitation fluorescence/coherent anti-Stokes Raman scattering (TPEF/CARS)

## Abstract

Long-term, repeatable monitoring of the appearance and progress of Alzheimer’s disease (AD) in real time can be extremely beneficial to acquire highly reliable diagnostic insights, which is crucial for devising apt strategies towards effective AD treatment. Herein, we present an optimized innovative cranial window imaging method for the long-term repeatable imaging of amyloid-β (Aβ) plaques and vessels in an AD mouse model. Basically, two-photon excitation fluorescence (TPEF) microscopy was used to monitor the fluorescently labeled Aβ plaques, whereas the label-free blood vessels were studied using coherent anti-Stokes Raman scattering (CARS) microscopy in the live in vivo AD mouse model. It was possible to clearly observe the Aβ deposition and vascular structure in the target cortex localization for 31 weeks in the AD mouse model using this method. The combined TPEF/CARS imaging studies were also instrumental in realizing the relationship between the tendency of Aβ deposition and ageing. Essentially, the progression of cerebral amyloid angiopathy (CAA) in the AD mouse model was quantitatively characterized, which revealed that the proportion Aβ deposition in the unit vessel can increase from 13.63% to 28.80% upon increasing the age of mice from 8 months old to 14 months old. The proposed imaging method provided an efficient, safe, repeatable platform with simple target localization aptitude towards monitoring the brain tissues, which is an integral part of studying any brain-related physiological or disease conditions to extract crucial structural and functional information.

## 1. Introduction

Careful monitoring of the cerebral cortex structures, such as neurons, glial cells, and blood vessels, in live in vivo models is indispensable for astutely studying every neurological disease. Alzheimer’s disease (AD) is an irreversible, virtually incurable neurodegenerative disorder that may eventually become terminal, and is the third highest mortality worldwide (just behind the reported deaths caused by cardiovascular diseases and cancer). Even though the precise causes and mechanisms of AD have not yet been resolved resolutely, the deposition of amyloid-β (Aβ) peptide has been identified as the indisputable hallmark of AD [1]. Aβ peptide is hydrolyzed from the Aβ precursor protein (APP), triggering the secretion of Aβ in cells, which can be precipitated and accumulated in the cell matrix to induce strong neurotoxicity. The neurotoxic effects of Aβ play a major role in the progression of AD. In 1991, Kowall et al. reported that the extent of tissue necrosis, loss of peripheral neurons and hyperplasia of nerve keratin caused by the injection of Aβ into the cerebral cortex of a mouse is directly correlated with the doses of Aβ administered [2]. The Aβ toxicity can also adversely affect the morphology and function of blood vessels to cause amyloidosis of the wall of blood vessels and enable vascular sclerosis, poor vascular elasticity, and thrombosis. In this way, it can induce premature apoptosis of nerve cells and promote the deposition of peripheral fibers to eventually result in the cerebral amyloid angiopathy (CAA). The role of Aβ fibrillation in AD pathogenesis has been well-recognized in different studies that accepted that curbing the Aβ burden holds the key towards achieving effective AD treatment [3,4]. Detailed investigations also confirmed that the growth of Aβ plaques is closely related to the size of Aβ plaques and the age of the AD-affected species [5,6,7]. For instance, Bolmont et al. found that small- and medium-sized plaques took a period of 4 weeks to grow, wherein a few plaques even exhibited size-shrinking [5]. Meyer-Luehmann et al. [6] reported that Aβ plaques can rapidly appear from monomeric Aβ peptides within 24 h, and can even remain stable in size for the next 14 days, as confirmed by imaging studies. Yan et al. [7] also monitored the growth in the size of Aβ plaques at different time intervals (7, 28, and 90 days). Therefore, devising new microscopic setups capable of long-term observation of Aβ deposition in an in vivo AD model could be highly advantageous to further understand the development and pathogenesis of AD with exclusive mechanistic detail, which will open up new avenues towards expediting the effective diagnosis and therapy of AD.

The feasibility of long-term in vivo brain imaging studies is highly dependent on the availability of reliable imaging techniques and/or labeling methods. In this regard, two-photon excited fluorescence (TPEF) microscopy, as an advanced optical technique, has been extensively employed in a vast array of in vitro/in vivo imaging applications, wherein the near-infrared femtosecond laser is often used as the light source. In comparison with single-photon fluorescence microscopy, it can offer deeper imaging depth, enhanced imaging contrast, and higher spatial resolution, as well as ensuring lower photo-damage and better resistance to the photobleaching; these characteristics make it an outstanding choice for the imaging of thick, living tissue samples. As a result, in recent years, TPEF microscopy has been successfully employed in a wide range of biomedical studies, including the potential diagnosis of many fatal diseases [8,9,10,11]. Coherent anti-Stokes Raman scattering (CARS) microscopy is another non-invasive advanced imaging technology that has been widely used in biochemistry, pharmacology, neurobiology, and other fields, as this label-free imaging method is advantageous for the imaging of biologically important macromolecules [12,13,14,15] such as proteins, lipids, nucleic acids, and carbohydrates [16,17,18,19,20]. Since blood vessels are mainly composed of lipid- and protein-rich endothelial cells, we envisage that CARS can also be potentially implemented to acquire the label-free long-term imaging of blood vessels [21].

In the in vivo mouse model, craniotomy is frequently performed to effectively monitor the mouse brain through different imaging techniques. The cerebral cortex, the prime area of interest for most of the in vivo brain (mouse model) imaging studies, usually remains covered, with the skulls of varying thickness (with the range of 100 µm–1 mm); this can jeopardize the bioimaging process by causing severe light scattering and the attenuation of signal intensity [22]. In order to improve imaging quality, usually a portion of the skull is surgically removed very carefully during the brain imaging studies to expose the cerebral cortex of the mouse brain, which is often replaced with transparent glass. However, the long-term exposure of the cerebral cortex to the air during the imaging studies with invasive craniotomy may not only impose an increasing risk of infection, but also can endanger the long-term acquisition of high-quality images, since the internal turbidity of the cranial window, caused by the likely inflammation, would adversely impact the output imaging signals emitted from the blood vessels [23]. Therefore, in craniotomy-based mouse models, maintaining a stable physiological environment at the imaging region is crucial to achieve long-term in vivo imaging/monitoring of the mouse brain. Additionally, to investigate the progression of AD in a living mice model, it is vital to study the same Aβ plaques for a long period of time, which requires an imaging system with the ability for long-term relocation and observation. However, the traditional imaging methods, wherein the Aβ plaques of interest need to be manually searched, are not conducive to monitoring the long-term dynamics of Aβ plaques. Yan et al. [7] monitored the growth in the size of Aβ plaques with a maximum interval of 90 days. Employing an extra image-locatable device to the traditional microscopy might be helpful for performing these studies, but this may not be cost efficient. In this context, Hefendehl et al. [24] developed an improved imaging setup, capable of rapidly relocating the previously marked areas automatically during the imaging process, using multiphoton in vivo imaging to study Aβ plaque formation in the brains of 3- to 4-month-old APP/PS1 transgenic mice over a period of 6 months. Bolmont et al. [5] also utilized a custom software that could record the location of two recognizable brain portions based on the relative positions of window borders and underlying blood vessels with a maximum interval of 1 month. Notably, it is almost impossible to observe Aβ plaques and the peripheral blood vessels simultaneously using the traditional microscopic devices described, owing to the post-staining difficulty in perceiving the dynamic changes in blood vessels. Therefore, it is highly important to smartly implement the simple imaging device(s) that could enable the real-time monitoring of the dynamics of Aβ plaques and the blood vessels at the same time, wherein the imaging of blood vessels requires no traditional labeling.

In the present study, we proposed a combination of TPEF/CARS imaging platforms along with a new cranial window method to simultaneously monitor the long-term Aβ deposition profile as well as the blood vessel dynamics in the brain in a craniotomy-based AD mouse model. Essentially, we not only employed a cranial window glass, composed of specially customized cranial glass window and hollow titanium ring to improve the airtightness, but also optimized the craniotomy technique to avoid the occurrence of glue infiltration and inflammation, so that a stable physiological environment is maintained in the cranial window to perform long-term imaging experiments without any fear of deteriorating health issues of the subject species. Furthermore, the advantages of employing label-free CARS imaging, coupled with a customized coverslip designing strategy (used as a superior cranial window glass), leveraged the scope of acquiring highly locatable imaging, wherein the relocation of multiple regions can be precisely determined throughout the imaging session. Essentially, the combined TPEF/CARS microscopy with certain considerate modifications in the imaging setup expedited the repetitive long-term observation of Aβ plaques and blood vessels at the specified locatable portions in the brain in a living in vivo mice model.

## 2. Materials and Methods

### 2.1. Animals

To perform the in vivo imaging studies in the AD mice model, 7-month-old male transgenic (Tg) APPswe/PS1dE9 (APP/PS1) [25] mice and their wildtype (WT) littermates were selected; these mice, with overexpression of human mutant amyloid precursor protein (APPV717F), are prevalently employed as the animal models of AD. All experimental mice were obtained from the Guangdong Provincial Animal Experiment Center of China. The experiments with the animals were performed as per the universally accepted standard guidelines, which were further reviewed and approved by the Experimental Animal Ethics Committee of Shenzhen University (No: A20220616, date: 9 March 2022).

### 2.2. Mouse Cranial Window Fabrication

The mice were first deeply anesthetized with 1.5–2% isoflurane, and the head of each mouse was immobilized with a specialized head immobilization device to perform the craniotomy. During the course of lengthy craniotomy-based operation, 100 µL of glucose was intraperitoneally injected into each mouse to replenish the energy and improve the survival rate of the mice. Vaseline was applied not only to protect the eyes of the mice, but also to prevent dehydration. With the help of a razor and depilatory cream, the hair from the head of each mouse was removed to expose the bare skin, and the skin was then carefully cut with a scalpel. The fascia on the surface of the skull along with the adjacent muscles were surgically removed to improve the adhesion of the mouse cranial window to the skull. As soon as the above operation was concluded, the skull was immediately moistened with a hemostatic sponge, soaked in normal saline to maintain a normal physiological state.

**Figure 1:**
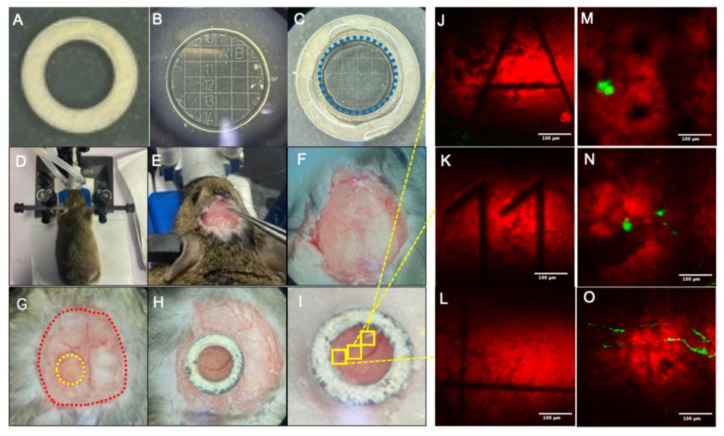
Fabrication and placement process of cranial window: (**A**) Customized hollow titanium ring. (**B**) Customized cranial window glass. (**C**) Cranial window assemblies bonded with optical glue; the blue dotted line represents the glass outline. (**D**) Head immobilization. (**E**) Fascia removal. (**F**) Preliminary processing completed. (**G**) Craniotomy, the yellow dotted line indicates the craniotomy area and the red dotted line indicates the exposed area of the skull. (**H**) Installation of cranial windows. (**I**) Bonding cranial window. (**J**–**L**) Coherent anti-Stokes Raman scattering (CARS) imaging of cranial window glass. (**M**–**O**) Mouse brain imaging of the corresponding position of cranial window glass. Scale bar: 100 µm.

Next, the normal saline was removed with cotton swabs to ensure the skull surface was completely dry. A toothpick dipped in 2-nitrile-acrylic acetate binder was then employed to evenly glue the scalp to the skull around the marginal non-cranial window area (red dotted line in Figure 1G). Sealing up the scalp and the skull not only can prevent bleeding or leakage of tissue fluid, but also can be effective in maintaining the firmness of the cranial window bonding. Selecting cranial window glass of apt size is crucial to perform stable long-term in vivo studies, since the use of cranial window glass too small in size will limit the imaging area, whereas using too large cranial window glass will not be able to fully fit with the curved skull surface, and result in compromised airtightness. Therefore, a cranial window glass with a diameter of 3 mm was selected to replace the skull, in order to perform the imaging. The craniotomy area is shown in Figure 1G, and the area, marked with yellow circle, is the position of the cranial window for this operation. The red dotted line indicates the margin where the scalp and the skull were sealed with the binder. Before grinding the bone, it is necessary to preliminarily grind the cranial window outline according to the size of the cranial window glass. The cranial window size is continuously compared during the grinding process to ensure the cranial window size is appropriate. The shape of the completed cranial window should be a standard circle with a diameter of the outer side corresponding to the diameter of the cranial window glass.

Due to the uneven distribution of Aβ plaques in the brain and their variations in shape, size, and location, it is extremely difficult to construct an apt imaging setup to perform long-term localizable imaging studies of Aβ plaques in the live in vivo model. Herein, the advantages of CARS microscopy in acquiring label-free imaging were intelligently exploited in designing an innovative coverslip as a cranial window glass that can be used for the efficient acquisition of localizable imaging, as shown in Figure 1B. The selected cranial window glass with a 500 µm grid scale was engraved with letter or number marks (A–U; 1–20) inside. The cranial window glass had a thickness of 170 µm. Due to the CARS microscopy, one can obtain the information of the cranial window glass in label-free imaging. When we observe the plaque of interest (Figure 1M–O), we can adjust the focal plane to the cranial window glass and record the number or letter information marked on the glass surface (as shown in Figure 1J–L, clearly depicting the edges of the grid and the numbers and letters in the grid), so that in the succeeding images, the Aβ plaque can be located by finding the corresponding position of the cranial window.

Owing to the strong bio-histocompatibility of titanium, a hollow titanium ring with an inner diameter of 2.5 mm and an outer diameter of 4 mm was designed to fix the cranial window. The hollow titanium ring and the cranial window glass was glued with Nolan optical UV adhesive (NOA81 optical UV adhesive, Norland, FL, USA). Fixing the titanium ring with the glass not only helped to maintain close contact between the cranial window glass and brain tissue, but was also beneficial in evading the breakage of the cranial window glass during subsequent placement, along with decreasing the degree of glue infiltration. After placing the cranial window glass in the area of the craniotomy, the titanium ring cranial window was gently pushed with forceps to ensure that the glass is completely embedded in the skull. After the titanium ring was placed against the skull, it was lightly pressed by forceps so that it could hold the cranial window glass tightly. Then, the skull surface around the titanium ring was filled with dental cement. The fabrication and placement process of the cranial window glass are effectively illustrated in Figure 1. After the surgery, the animals were allowed to recover for 1 week before starting the imaging process.

### 2.3. In Vivo TPEF/CARS Imaging

**Figure 2:**
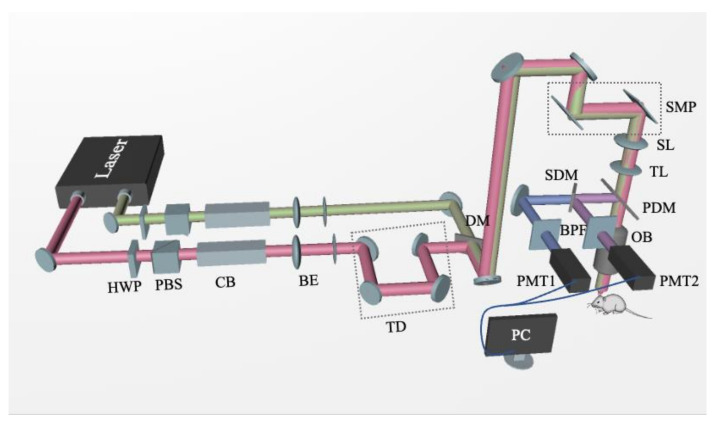
Schematic diagram of two-photon excitation fluorescence/coherent anti-Stokes Raman scattering (TPEF/CARS) system: Laser, Ti:Sapphire dual-output solid-state laser; HWP, half-wave plate; PBS, polarization beam splitter; CB, chirped block; BE, beam expander; TD, time delay; DM, dichroic mirror; SMP, scanning mirror pair; SL, scanning lens; TL, tube lens; PDM, primary dichroic mirror; OB, objective lens; SDM, secondary dichroic mirror; BPF, bandpass filter; PMT, photomultiplier tube.

Mice were intraperitoneally injected with Methoxy-X04 (5 mg/kg; dissolved in 10% vol DMSO and 90% vol PBS) for performing the in vivo TPEF imaging of Aβ plaques. After 24 h, mice were anesthetized with isoflurane (maintained at 1.5–2% throughout imaging) and the head of each mouse was immobilized using a head immobilization device. Schematic representation of the imaging system, used for performing the experiments, is shown in Figure 2. To perform CARS imaging, a similar procedure, detailed in our previous report, was followed, which investigated the changes in lipid profile in the AD mouse brain tissue [26]. Essentially, for TPEF/CARS imaging, a femtosecond (fs) laser (Chameleon Discovery, Coherent Inc. Santa Clara, USA) with two outputs was used as the light source, wherein the Stokes beam was 1040 nm (pulse width 100 fs) and the pump beam was 800 nm (pulse width 100 fs). The pump light beam was set at 800 nm not only to match the vibration of CH_2_ in lipids, but also to excite the two-photon excited fluorescence of the fluorescent dye methoxy-X04. The spectral resolution was improved by introducing the linear chirp of the high-dispersion glass rod. The Raman spectra of different wave numbers can be obtained by changing the optical path difference of two laser beams through a time delay device. After the two laser beams are combined, laser passes through the galvanometer scanning system, and is focused onto the sample by an objective lens. The TPEF/CARS signal generated by the sample can be collected by the same objective, separated by the dichroic mirrors and band pass filters; which finally can be detected by the PMTs. The output power of the pump light is around 30 mW and the output power of the Stokes light is around 80 mW. The Raman spectra were measured in the range from 2800 to 3000 cm^−1^, with an integration time close to 10 s. The imaging depth was about 200 µm, and the field of view was 410 × 410 μm^2^ under a 20× water immersion objective. The spatial resolution of the system was about 470 nm, and the spectral resolution was 18 cm^−1^.

### 2.4. Image Analysis

ImageJ Software was used to convert the TPEF images of the plaques into 8-bit grayscale images with appropriate thresholds to analyze the longitudinal changes in the cross-sectional area of individual plaques. All detected plaques were manually reviewed, and if they were located on the edge of the thinned-skull window, or exhibited a fluorescence intensity less than the mean intensity of an adjacent background region, or the images were affected by motion artifacts (from heartbeat or respiration), they were deemed unreliable and excluded from analysis.

## 3. Results

### 3.1. In Vivo Imaging of Aβ Plaques and Blood Vessels

**Figure 3:**
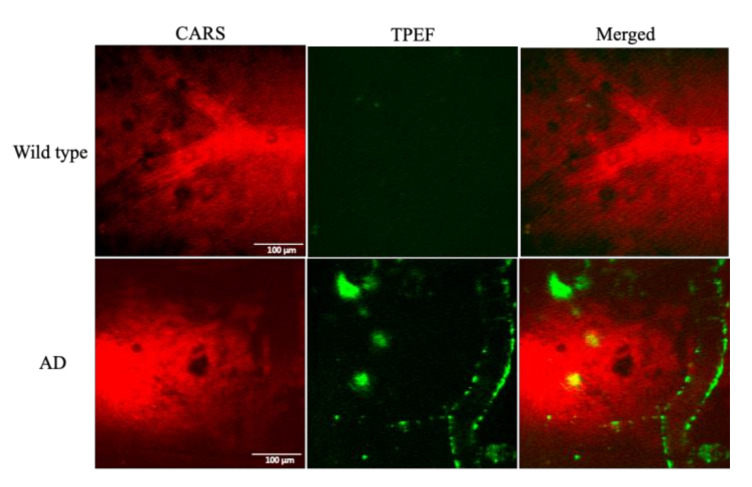
TPEF/CARS images of Aβ in the brain of Alzheimer’s disease (AD) mouse model: (**left**) CARS images at 2885 cm^−1^; (**middle**) 800 nm excitation TPEF images; (**right**) merged images. Scale bar: 100 µm.

To perform the in vivo imaging studies of Aβ plaques, 7-month-old APP/PS1 mice and their wildtype (WT) littermates were subjected to the rationally designed TPEF/CARS microscopy. The images, obtained from the TPEF and CARS microscopic studies, along with their merged images, are shown in Figure 3, which clearly reveals that the selected region of the cerebral cortex of the AD mouse brain can be effectively imaged using the combination of TPEF/CARS microscopic techniques. Basically, the CARS images of C-H vibration were instrumental in perceiving the blood vessels, wherein even the blood flow was conspicuous. On the other hand, methoxy-X04-labeled Aβ plaques and CAA can be clearly distinguished from the background using TPEF imaging. When the two images are merged, visualization of the label-free blood vessels and the deposition of fluorescently labeled Aβ plaques, signifying the relative position of Aβ plaques on the vascular wall, becomes feasible. It is evident from the imaging results that the WT mice group did not reveal any sign of Aβ deposition, whereas the deposition of Aβ plaques in the cerebral cortex of AD mouse model is indisputable.

### 3.2. Long-Term Visualization of Amyloid Plaques

**Figure 4:**
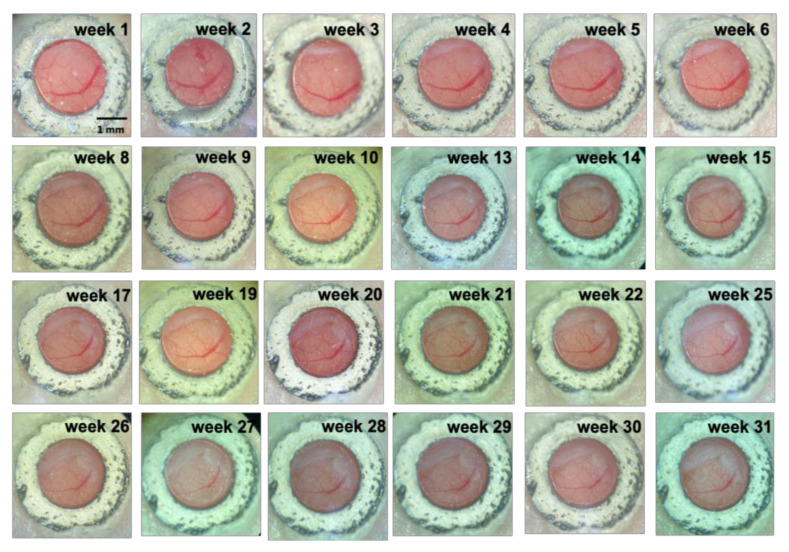
Visualization of cranial window over time. Scale bar: 1 mm.

**Figure 5:**
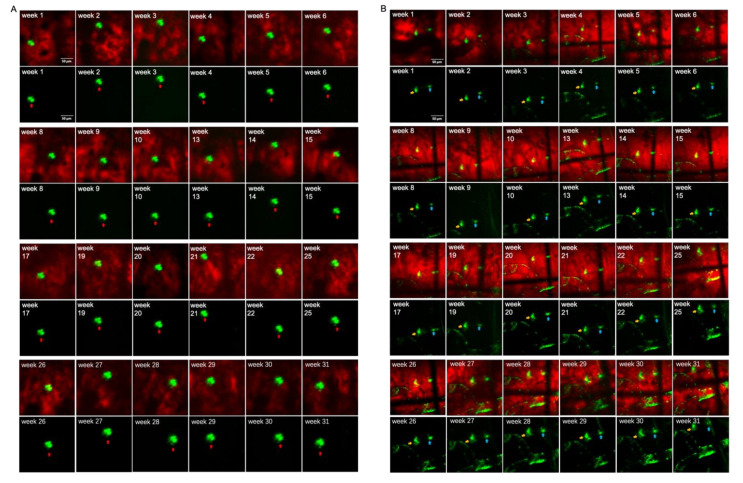

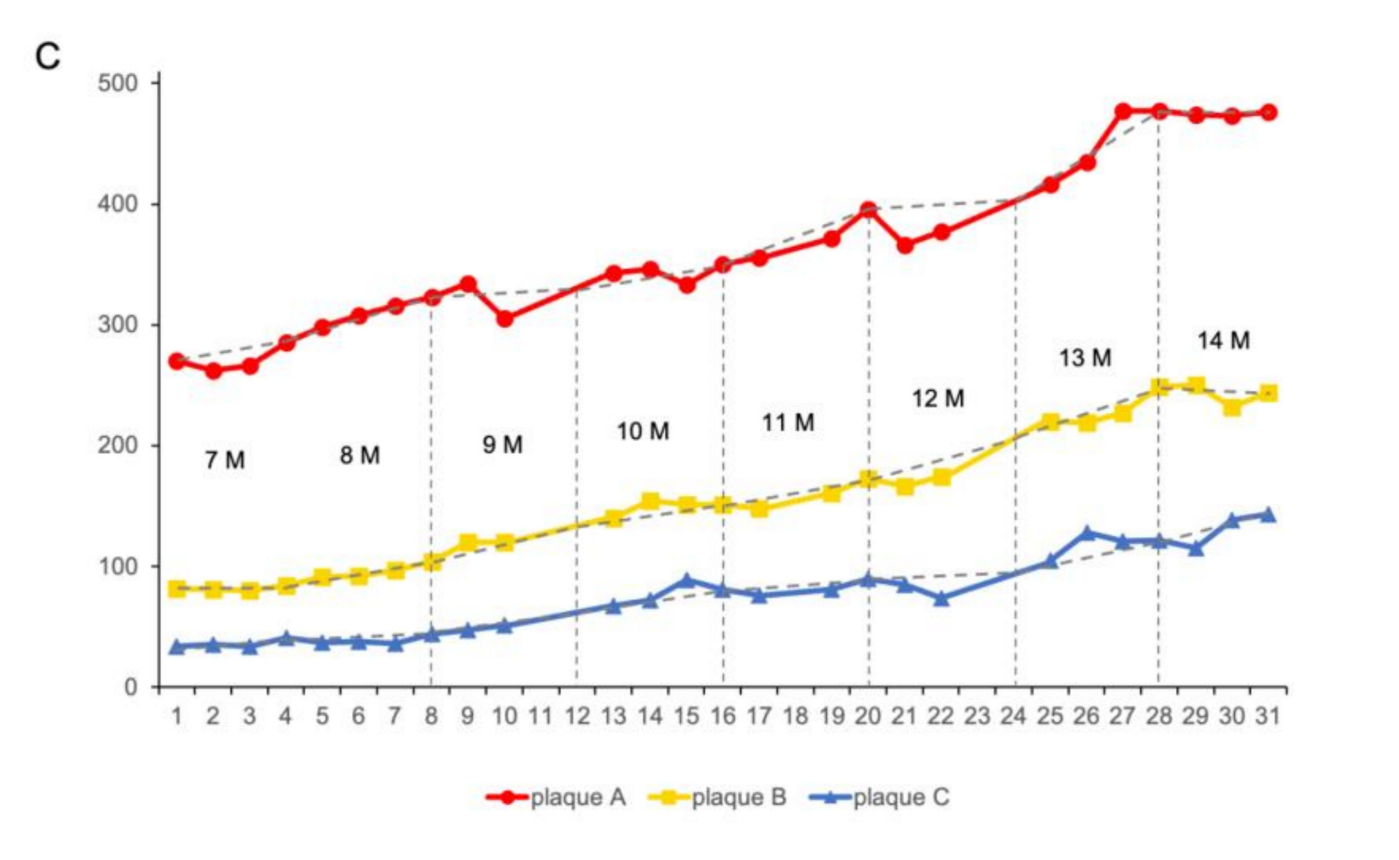
Aβ plaques grow over time: (**A**) Plaque A (red arrow) was imaged for up to 31 weeks. (**B**) Plaques B (yellow arrow) and C (blue arrow) were imaged for up to 31 weeks. Scale bar: 50 µm. (**C**) Plaque A, B, C cross-sectional areas are shown over time.

Using TPEF/CARS microscopy, Aβ plaques in the individual AD mouse models were imaged through skull cranial windows, wherein the 7-month-old mice were imaged at different time intervals over a period of 31 weeks (Figure 4). During imaging, since the thickness of the plaque may also vary slightly, the maximum cross-section of the fluorescent plaque was selected as the focal plane for statistical analysis to ensure the consistency of the data. The microscopic studies clearly demonstrate that there was an increasing accumulation of Aβ plaques with extended time intervals over a period of 31 weeks, and the cross-sectional area of each of the three studied plaques (denoted as A, B, and C) had undergone about 76.29%, 197.56%, and 320.58% of overall growth, respectively, after 31 weeks. As evident in Figure 5, initially, the 7-month-old mice underwent negligible change in the size of the three Aβ plaques for the first 4 weeks. However, from the 5th week onwards (the mice became 8 months old), all the plaques started showing a stable upward trend in their size growth. Eventually, when the mice became 10 months old or older (11-, 12- or 13 months old), our analysis revealed a rapid growth in the size of each Aβ plaque, wherein the growth rate of plaque C (Figure 5B, blue arrow) was usually found to be higher than that of the plaque A (Figure 5A, red arrow) and B (Figure 5B, yellow arrow). The highest monthly growth rate for plaque C reached 34.21% when the mice became 11 months old (Table 1). Moreover, once the age of the studied mice reached 10 months, the amyloid deposition on the vascular wall became conspicuous. Upon further ageing of the mice, vascular amyloid deposition underwent a rapid accumulation, which significantly aggravated the CAA phenomenon. Once the mice turned 14 months old, the size of the plaques became stable, apparently showing insignificant change in size. Here, the trends in the growth of three plaques also indicate that the rate of size growth also depends on the initial plaque size, wherein the smaller plaques tend to exhibit higher growth rate compared to the larger plaques, as already reported by Yan et al. [7].

**Figure 6:**
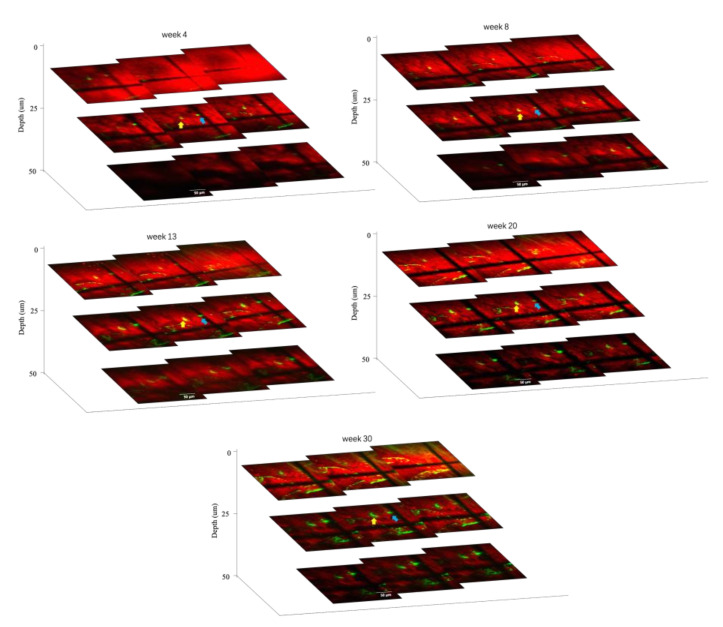
Plaques B (yellow arrow) and C (blue arrow) imaged at different depths over 4-, 8-, 13-, 20-, and 30-week intervals. Scale bar: 50 µm.

In addition, with the same AD mice model, TPEF/CARS imaging of Aβ plaques B and C were performed at different tissue depths (with a gap of 5 µm) to ascertain the deviations in the volume and morphology for each plaque with time (4, 8, 13, 20, and 30 weeks). As evident in the Figure 6, the thicknesses of plaques B and C, which were ~15 µm and ~10 µm, respectively, at 4th week experienced a slight increase in the cross-sectional area and thickness upon reaching the 8th week, and this was accompanied by the emergence of mild CAA. A significant increase in the plaque size and thickness was observed for every tissue depth upon comparing the merged images after the 13th week with those of the images obtained after 20th week. Finally, the imaging studies revealed that after 30 weeks, the thicknesses of the plaques B and C at 30 µm and 25 µm underwent almost 100% and 150% growth, respectively. These results also reiterate the faster growth of smaller plaques over the larger plaques. Therefore, the overall imaging results confirm that the individual in vivo growth of amyloid plaques is directly correlated with animal age and plaque size.

### 3.3. Visualization of CAA over Time

**Figure 7:**
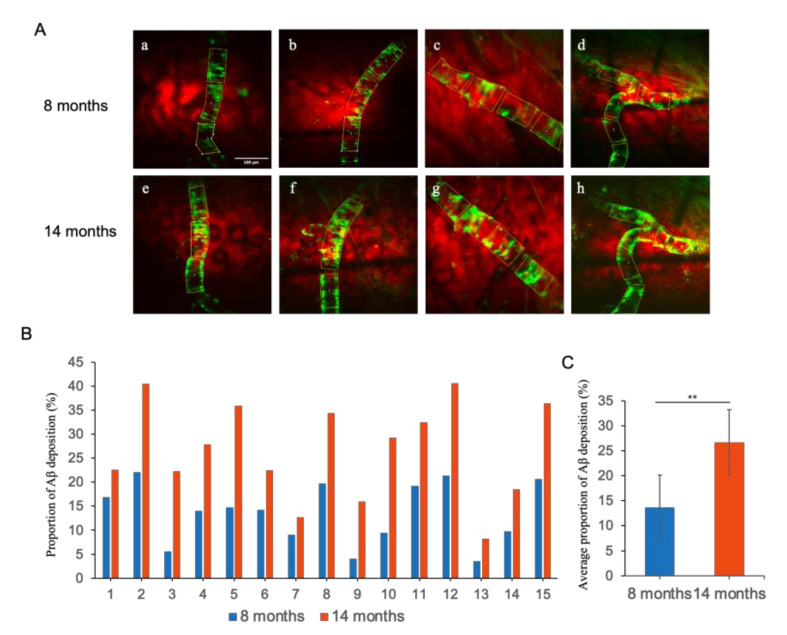
(**A**) CAA in the brains of 8-month-old (**a**–**d**) and the same location in 14-month-old (**e**–**h**) AD mouse models. Scale bar: 100 µm. (**B**) The percentage (%) of Aβ deposition in 15 locatable per unit length vessels in 8-month-old and 14-month-old mice. (**C**) Average percentage (%) of Aβ deposition. Data represent mean ± SD from independent replicates. Asterisks denote significance compared with 8 months group and 14 months group (** *p* < 0.01, *t*-test).

In order to obtain a quantitative appraisal of the ageing related progression of CAA in AD mice model, the average of the fluorescently labeled areas, denoting vascular Aβ deposition, were measured for both 8- and 14-month-old mice, respectively. As shown in Figure 7A, a comparatively lower amount of Aβ deposition was detected in the perivascular space of the cerebral cortex region of the 8-month-old mice, whereas the 14-month-old mice group endured significantly higher Aβ deposition. To calculate the percentage of the fluorescently marked area in the unit vessel, a length of 100 µm was considered as the unit vessel length (as shown in the yellow box in Figure 7A). In total, 15 locatable fluorescently labeled areas were selected for quantitatively realizing the proportion of Aβ deposition, wherein each specified area represented a unit length vessel. The proportion of Aβ depositions were in the range of 4.04% to 21.38% for the different specified fluorescently labeled areas in the case of 8-month-old mice (Table 2), which accounts for about 13.63% Aβ deposition per unit vessel length area on average (Figure 7B), and it can be considered as mild CAA. In 14-month-old mice, the proportion of Aβ depositions in 15 different unit vessel length selected areas were in the range of 8.19% to 40.61% (Table 2), revealing about 26.69% Aβ deposition per unit vessel length area on average (Figure 7B), which can be characterized as severe CAA. Therefore, an increasing deposition and subsequent accumulation of Aβ in the blood vessels were observed in the AD mice model upon ageing, which is consistent with our previous observations. However, this experiment concerning the analysis of the Aβ accumulation and the progression of CAA was unable reiterate whether the vessels with lesser Aβ deposition underwent faster accumulation than those with higher Aβ deposition, perhaps owing to the differences in plaque accumulation and CAA mechanisms.

## 4. Discussion and Conclusions

Long-term in vivo imaging of Aβ plaques is extremely important to understand Aβ dynamics, which has great significance in devising new strategies towards effective diagnosis and treatment of AD, a chronic neurodegenerative disease. In the present study, we monitored the dynamic growths of Aβ plaques in the live in vivo mouse model over a period of 31 weeks using the combined TPEF/CARS microscopic techniques. Essentially, by employing a novel cranial window method, reliable detection of the accurate location of previous imaging positions became feasible, which negated the requirement of implementing any blood vessel markers for simultaneously monitoring the Aβ plaques and vessels in AD model.

Over a period of 31 weeks, an increasing accumulation of the Aβ plaques was observed. In the three selected Aβ plaques (A, B, and C), the gradual incremental growth in size was correlated with ageing, wherein the growth rates for the cross-sectional areas of the three plaques were calculated to be 76.29%, 197.56%, and 320.58%, respectively. It was evident that the growth rates of the plaques in APP/PS1 mice were highest at the age range of 10–13 months; the plaques underwent a significant decrease with further ageing of the mice. This result is consistent with other reports, which either revealed minimal Aβ plaque growth in the case of extremely old mice [27], or documented no obvious saturation in the growth of Aβ plaques in the 4–9-month-old APP/PS1 mice [24]. Consistent with these findings, researchers also reported a key relationship between Aβ concentration in interstitial fluid and Aβ plaque growth [7]. Therefore, it can be implied that in presence of excess soluble Aβ, the Aβ plaques can endure continuous layer by layer addition of the Aβ peptide to the existing plaques, resulting in aging-related exponential growth in size. However, the sharp growth in the Aβ deposition with aging is sustainable up to a certain point, and the rate of the growth of Aβ plaques is expected to slow down at an older age owing to the inability of the system to meet the exponential demand of soluble Aβ, which needs to be incorporated into the plaques to maintain the growth rate. In addition, our results also show that the smaller plaques can exhibit a higher growth rate compared to the larger plaques, which was conclusively proven by the thickness-dependent imaging studies.

To visualize CAA, the proportions of Aβ deposition per unit length of blood vessels were systematically imaged for 8- and 14-month-old AD mice. Analyzing the CAA imaging results over a period of 6 months revealed that a mild CAA is present in the 8-month-old mice with an average Aβ deposition rate of 13.63%, whereas the average rate of Aβ deposition was 26.69% in the case of 14-month-old mice, indicating severe CAA in the brain. An incremental accumulation of Aβ in the blood vessels, indicating elevated CAA upon aging, can be regarded as the gradually raising symptoms of AD, which highlights the aging-related progress of AD in the mice model. However, unlike the case of Aβ plaques, which underwent a size-dependent growth in size (smaller-sized plaques endured faster growth), the frequency of Aβ deposition in the blood vessels in the case of CAA did not show any direct correlation with the initial amount of already accumulated Aβ, which may be due to their fundamental differences in the Aβ accumulation mechanisms. It is worth mentioning that an increasing level of deposition of Aβ plaques in the brain resulted in the declining of Aβ clearance. Although there is lack of lymphatic vessels in the brain, recent studies have shown that the lymphatic drainage in the brain mostly occurs through the perivascular space (Virchow–Robin space, VRS) [28,29,30]. During the process of Aβ clearance through VRS drainage, insoluble Aβ fibers are likely to be deposited gradually in the cortex and around the subpial meningeal arterioles (mainly the vessel wall and astrocyte glial limiting membrane), which facilitates the formation and growth of localized Aβ plaques to invade the arterial wall, resulting in CAA. It is worth mentioning that the pulsation of cerebral arteries is the driving force of the Aβ clearance through VRS. Therefore, in AD patients, once the Aβ plaques are formed on the cerebral artery walls, it significantly weakens the pulsation of cerebral arteries, which in turn restricts the clearance of the aggravated Aβ to promote the accumulation of Aβ fibers on the cerebral artery walls [31]. In addition, the formation of Aβ plaques can also block VRS, which may cause cerebral lymphatic circulation disorders to further endorse the deposition of Aβ fibers on the walls of cerebral arteries, leading to a vicious circle. Therefore, in the present report, studying the age-related appearance and development of CAA quantitatively in the in vivo mouse model using a newly developed cranial window method not only provided a clear mechanistic insight into AD, but also highlights the scope of further studying the growth of surrounding Aβ plaques to understand their role in the progress of AD.

In summary, in vivo TPEF and CARS imaging can be useful to evaluate the distribution of Aβ in cortex of brain, and to analyze the age-dependent appearance and growth of Aβ plaques in APP/PS1 transgenic AD mice. A rationally designed novel cranial window method enabled the determination of the accurate localization of previous imaging positions reliably, without the need of employing an external blood vessels marker. This method provided a solid foundation towards understanding the appearance, development, and pathogenesis of Aβ plaques to expedite the development of effective diagnosis and treatment for AD.

## Figures and Tables

**Table 1:** The growth rate (%) of plaques A, B, and C per month from 7-month-old to 14-month-old mice.

Growth Rate%/Month	7	8	9	10	11	12	13	14
plaque A	5.56	8.39	6.19	20.41	11.24	9.29	14.66	0.43
plaque B	2.44	14.28	12.91	7.86	16.89	26.51	13.18	−6.40
plaque C	2.94	5.41	27.65	20.9	34.21	23.53	16.19	24.35

**Table 2:** Aβ deposition rate (%) per unit vessel length in 8-month-old and 14-month-old mice.

	8 Months				14 Months			
	a	b	c	d	e	f	g	h
1	16.90	14.04	9.03	19.24	22.56	27.91	12.75	32.46
2	22.12	14.78	19.73	21.38	40.54	35.91	34.43	40.61
3	5.55	14.24	4.04	3.55	22.30	22.50	15.97	8.19
4	N/A	N/A	9.45	9.78	N/A	N/A	29.31	18.55
5	N/A	N/A	N/A	20.63	N/A	N/A	N/A	36.44

## Data Availability

Not applicable.
